# Multi-Stage micro-CT–based dimensional accuracy assessment of a voronoi-based patient-specific cranial implant fabricated by electron beam melting

**DOI:** 10.3389/fmedt.2026.1811963

**Published:** 2026-04-24

**Authors:** Horațiu Rotar, Cosmin Cosma, Nicolae Bâlc, Seong-Gon Kim, Daniel Ostaș

**Affiliations:** 1 Department of Oral and Maxillofacial Surgery and Implantology, Faculty of Dental Medicine, “Iuliu Hatieganu” University of Medicine and Pharmacy, Cluj-Napoca, Romania; 2 Department of Oral and Maxillofacial Surgery, Medicover Hospital Cluj-Napoca, Cluj, Romania; 3 Medicad, Oradea, Bihor County, Romania; 4 Additive Manufacturing and Rapid Product Development Research Center, Technical University of Cluj-Napoca, Cluj-Napoca, Romania; 5 Department of Oral and Maxillofacial Surgery, College of Dentistry, Kangwon National University, Gangneung-si, Republic of Korea

**Keywords:** additive manufacturing, cranioplasty, cranial implant, dimensional accuracy, electron beam melting, micro-computed tomography, patient-specific implant, Voronoi-based design

## Abstract

**Introduction:**

Patient-specific cranial implants fabricated using powder bed fusion technologies require rigorous dimensional validation to ensure reliable digital-to-physical translation, particularly when complex architectures are employed. However, multistage quantitative evaluations that distinguish deviations introduced during additive manufacturing and post-processing remain limited.

**Methods:**

A multistage dimensional accuracy assessment workflow based on high-resolution micro-computed tomography (micro-CT) was applied to a representative patient-specific cranial implant featuring a Voronoi-based macrostructure fabricated in Ti–6Al–4 V using Electron Beam Melting (EBM). The implant was digitized in the unpolished and surface-finished conditions. Three-dimensional surface models were compared with the original virtual design using full-field deviation analysis to quantify geometric variations attributable to manufacturing and post-processing.

**Results:**

After the finishing procedure, the surface roughness decreased significantly, with Ra reduced from 19.41 µm to 1.43 µm and Rz from 83.28 µm to 6.31 µm. A comparison between the virtual model and the unpolished implant revealed dimensional deviations predominantly within −0.20 to +0.35 mm, with localized positive deviations up to +0.55 mm associated with powder adhesion and support interaction. Following post-processing, deviations shifted predominantly toward negative values owing to controlled material removal, with a mean deviation of −0.05 mm and a maximum negative deviation of −0.18 mm. Surface finishing reduced the maximum positive deviation from +0.55 mm to +0.04 mm, corresponding to an approximate 93% reduction in positive dimensional deviation. The overall topology and structural continuity of the Voronoi-based architecture were preserved throughout manufacturing and finishing.

**Discussion:**

This proof-of-concept study demonstrated the feasibility of a multistage micro-CT-based workflow for the dimensional validation of complex patient-specific cranial implants fabricated by EBM. The proposed methodology enables differentiation between manufacturing- and post-processing-induced deviations and supports reproducible quality assessment of additively manufactured cranial implant geometries.

## 1 Introduction

Patient-specific cranial implants produced using additive manufacturing (AM) technologies have become an integral component of modern cranioplasty workflows, enabling the accurate restoration of complex cranial defects while facilitating improved intraoperative handling and potentially reducing operative time (1–7). Beyond anatomical congruence, the dimensional accuracy of these implants is a critical determinant of clinical performance, influencing implant fit, fixation stability, and risk of postoperative complications (3, 8, 9). Consequently, robust and quantitative validation of dimensional fidelity is an essential prerequisite for the clinical translation of additively manufactured cranial implants.

Powder bed fusion technologies, including Selective Laser Melting (SLM) and Electron Beam Melting (EBM), are widely employed for the fabrication of titanium cranial implants owing to their ability to produce geometrically complex, patient-specific components with favorable mechanical properties and biocompatibility (5, 7, 10, 11). However, the manufacturing process is inherently associated with dimensional deviations arising from thermal gradients, layer-wise consolidation, residual stresses, and the subsequent need for post-processing procedures, such as support removal and surface finishing (12–15). These deviations may be further amplified in implants featuring complex, non-periodic architectures, underscoring the necessity of a systematic dimensional accuracy assessment across multiple production stages.

Previous studies investigating the dimensional accuracy of cranial and craniofacial implants have frequently relied on optical scanning techniques or limited point-based measurements (9, 16). Although optical methods are suitable for relatively simple geometries, they may present notable limitations when applied to intricate lattice- or network-based structures, particularly in the presence of internal cavities, narrow openings, and reflective metallic surfaces, which may lead to incomplete surface capture and reconstruction artifacts (12, 13). In contrast, micro-computed tomography (micro-CT) enables the nondestructive volumetric digitization of complex metallic implants, providing highly comprehensive geometric information that can be converted into high-resolution surface models suitable for full-field dimensional analyses (10, 15).

Despite the increasing adoption of EBM for patient-specific cranial implants, studies presenting a multistage, full-field dimensional accuracy evaluation that quantitatively assess deviations introduced during additive manufacturing and subsequent post-processing are lacking. In particular, the combined use of micro-CT-derived surface models and dedicated metrology software for systematic comparison between the virtual design and manufactured implant remains underreported in the context of cranial reconstruction and medical-grade implant validation (9, 10, 15).

Previous work in which the authors were involved demonstrated the feasibility of producing a Voronoi-based patient-specific cranial implant using laser powder bed fusion, with a primary focus on design strategies and manufacturing feasibility (17). Although this prior study established the applicability of bio-inspired macrostructures in cranioplasty, a comprehensive quantitative validation of dimensional fidelity, particularly using Electron Beam Melting and high-resolution metrological techniques, has not yet been reported (17).

The present study addresses this gap by proposing and validating a methodological workflow for the dimensional accuracy assessment of an EBM-processed, patient-specific cranial implant featuring a previously described Voronoi-based architecture (17). Using a representative clinical case as a demonstrator, dimensional deviations were quantitatively evaluated at three critical stages: (1) between the original virtual design and the unpolished implant prior to post-processing, (2) between the original design and final post-processed (polished) implant, and (3) between the unpolished and polished implant. High-resolution micro-CT imaging and full-field deviation analysis were employed to characterize the geometric variations induced by the manufacturing and finishing processes. By focusing on dimensional fidelity rather than design novelty, this study aimed to provide a reproducible and clinically relevant validation framework for complex additively manufactured cranial implants fabricated via EBM, focusing on methodological validation rather than population-level accuracy benchmarking.

## 2 Materials and methods

### 2.1 Implant design

The patient-specific cranial implant evaluated in this study was generated using a previously established computer-aided design (CAD) workflow that incorporated anatomical reconstruction and Voronoi-based generative modeling. The complete design methodology, including medical image processing, anatomical surface reconstruction, and macrostructural generation based on Voronoi tessellations, has been described in detail elsewhere (17) and is not the focus of this paper.

**Figure 1:**
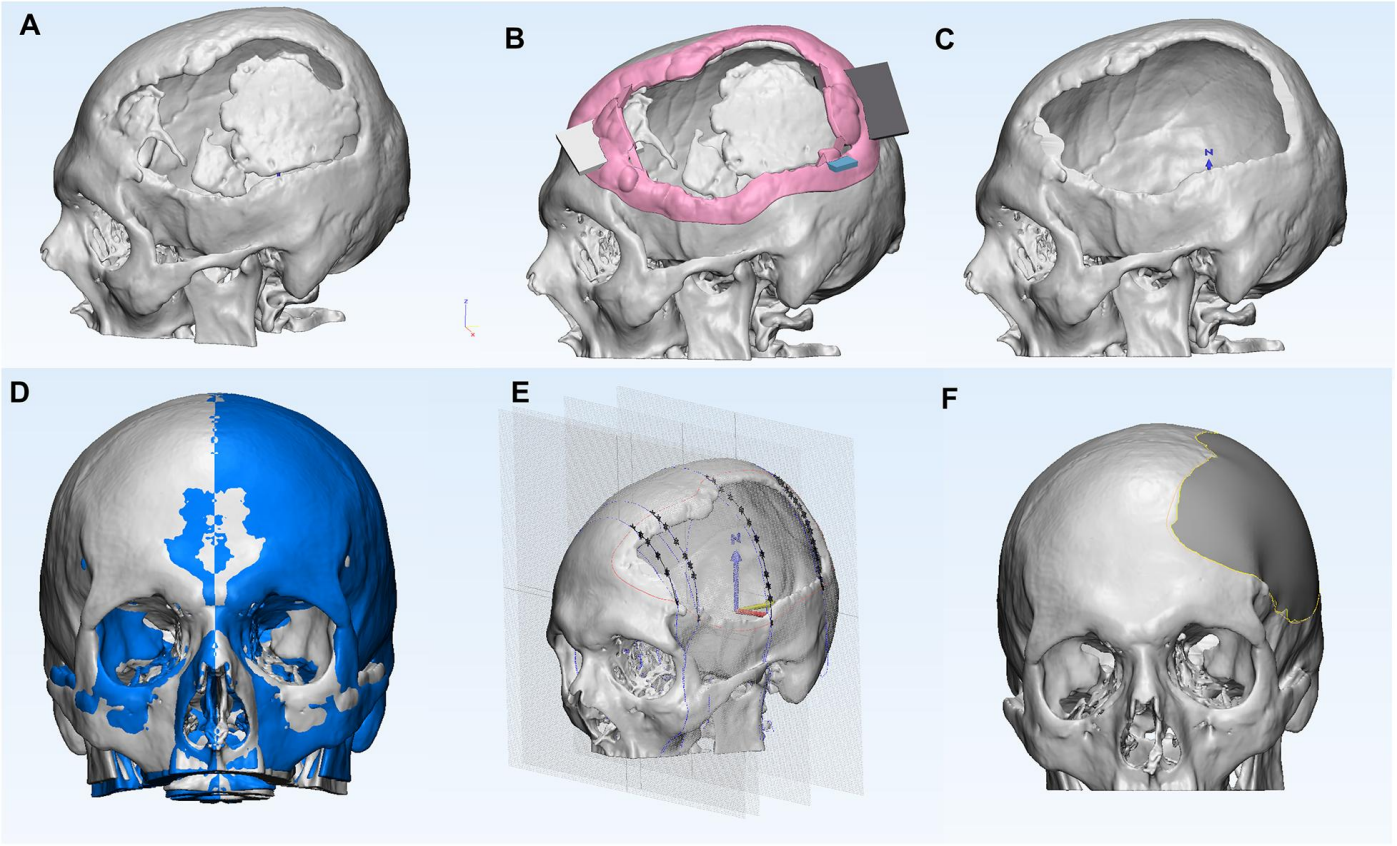
Schematic representation of the anatomical modeling phases of the Voronoi-designed cranial plate: **(A)** the initial anatomical model of the skull with floating bone fragments. **(B)** the anatomical model of the skull with the osteotomy guide for the floating bone fragments. **(C)** the anatomical model of the skull with the removed floating bone fragments, prepared for reconstruction. **(D)** creation of a median reference plane and mirror projection of the healthy part (blue color) onto the side with the defect. **(E)** sketches positioned perpendicular to the median reference plane and data acquisition from the reference (curve delimiting the defect, the original skull with the defect, and the mirrored skull) in sketches. **(F)** the reconstructed skull with the cranial plate - anatomically modeled plate.

Briefly, an anonymized computed tomography (CT) dataset of a unilateral fronto-temporo-parietal cranial defect was used to generate a three-dimensional (3D) anatomical skull model using Mimics Innovation Suite 26.0 (Materialise NV, Leuven, Belgium). The resulting anatomical model was exported to 3-matic 18.0 (Materialise NV, Leuven, Belgium), where a mirror-based reconstruction of the contralateral healthy side was performed to restore the missing cranial contour, followed by the generation of a patient-specific cranial plate exhibiting tangential overlap with the surrounding bone (Figure 1).

The external surface of the reconstructed plate served as the reference geometry for generating a Voronoi-based macrostructure characterized by an irregular, non-periodic network of interconnected struts and multiple openings.

**Figure 2:**
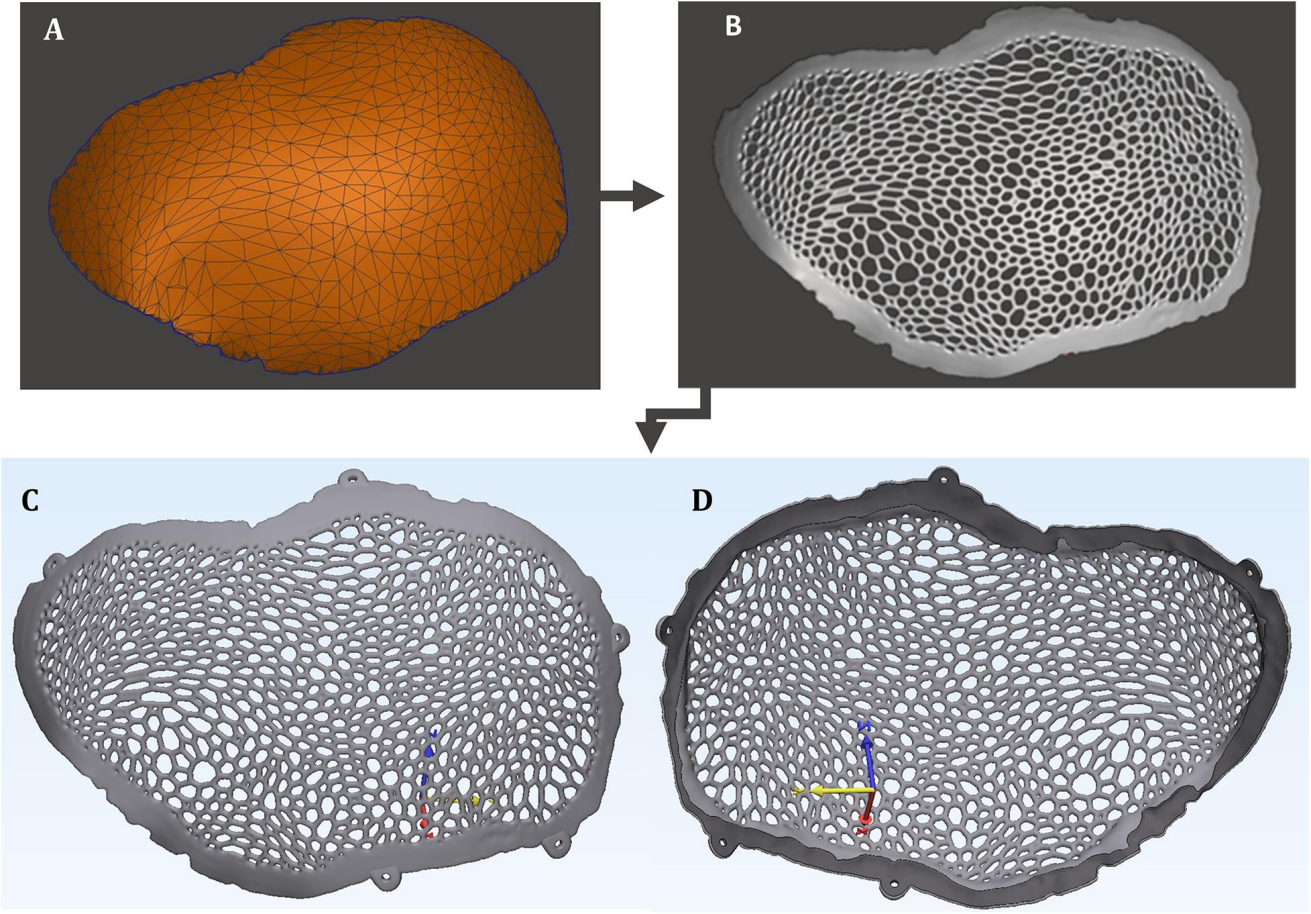
Generative modeling phase of the biomimetic patient-specific cranial implant: **(A)** simplified triangular mesh of the cranial implant. **(B)** biomimetic patient-specific cranial implant with Voronoi architecture. **(C)** Three-dimensional virtual model of the five-point bone fixation voronoi cranioplasty plate—external implant surface. **(B)** Three-dimensional virtual model of the five-point bone fixation Voronoi cranioplasty plate: internal implant surface.

Compared to the previously published study (17), several design modifications were implemented: (1) the Voronoi generative process was performed using the “Dual Edges” function in Meshmixer (Autodesk Inc., San Rafael, CA, USA) rather than the “Mesh + Delaunay Dual Edges” command; (2) the generative algorithm was applied exclusively to the isolated external surface of the cranial implant rather than the full-thickness stereolithographic (STL) model (Figures 2A,B); and (3) the fixation configuration was modified by incorporating five peripheral fixation flanges, replacing the four angular fixation points used in the previously reported Voronoi-based implant design (17) (Figures 2C,D).

The resulting model was subsequently imported into 3-matic 18.0 (Materialise NV, Leuven, Belgium) for the final modeling. At this stage, fixation flanges with predefined screw holes for osteosynthesis were integrated, and localized manual thickening was applied to the internal peripheral region of the implant. This step aimed to increase the implant–bone contact surface and facilitate accurate adaptation to the cranial defect following the application of the “Undercut Removal” function in the 3-matic software.

The anatomical modeling and generative design phases resulted in a Voronoi Dual Edges–type patient-specific cranial implant with a five-point fixation (Figures 2C,D).

As previously mentioned, in the present design five fixation flanges were incorporated instead of the four angular fixation points described in the previously published Voronoi-based cranial implant design (17). This modification was introduced primarily to adapt the implant to the size and geometry of the cranial defect evaluated in the present study. For relatively large cranial implants, the distribution of multiple fixation flanges along the implant periphery may improve fixation symmetry and overall implant stability during screw fixation. Additional clinical considerations also influenced the flange configuration. The positioning of fixation points was guided by regional variations in cranial bone thickness to ensure secure screw anchorage. Furthermore, particular attention was given to the frontal region, where limited hair coverage may increase the visibility of fixation hardware; therefore, flange positioning was selected to balance mechanical stability with aesthetic considerations related to flange relief visibility.

In the present study, the final virtual implant model was used exclusively as the nominal reference geometry for the subsequent dimensional accuracy assessment. No dimensional compensation was introduced at the design stage to account for material removal during post-processing.

### 2.2 Electron beam melting manufacturing

A patient-specific cranial implant was fabricated using the EBM process, an additive manufacturing technology suitable for producing complex metallic components under high-vacuum conditions. The EBM process is classified as a powder bed fusion (PBF) technology according to the ISO/ASTM 52900:2021 Additive Manufacturing standard. The manufacturing was performed by the industrial service provider FIT AG (Lupburg, Germany) using a Ti–6Al–4 V titanium alloy powder and an Arcam Q10 machine. The spherical titanium powder was obtained via gas atomization, with particle sizes ranging from 45 to 105 µm. The chemical analysis performed on the powder shows that the composition complies with surgical implants standards such as ISO 5832-3 and ASTM F1472. The results are presented in Table 1, and all measured values fell within the limits specified by these standards.

**Table 1:** Chemical analysis of Ti-6Al-4 V powder used in the EBM process.

Element	Min (%)	Max (%)	Result (%)
Al	5.50	6.50	6.42
V	3.50	4.50	4.13
Fe	–	0.30	0.18
O	–	0.20	0.142
C	–	0.08	0.01
N	–	0.05	0.02
H	–	0.015	0.003
Y	–	0.005	<0.001
Ti	balanced	balanced	balanced

The production workflow for a patient-specific cranial implant begins with the data preparation of the STL file, during which the optimal build orientation is determined. In this case, the component was fabricated in a vertical orientation. Based on this orientation, support structures were generated where necessary, and the model was scaled to compensate for thermal shrinkage during cooling. The STL file was then sliced into 2D layers, and the resulting slice file was transferred to the EBM system.

The implant was fabricated using the EBM process with a layer thickness of 50 µm, ensuring complete melting of the powder and strong interlayer bonding. The process was carried out using an optimized parameter set (Version 4.2.72) with automatic power calculation enabled, allowing dynamic regulation of beam current and energy input by the EBM control software depending on the scan length. In this configuration, longer scan lines were processed at higher beam speeds than shorter ones, ensuring consistent energy distribution. The selected process parameters included current compensation, thickness function (enabled), three contour passes, and a hatch line offset of 0.2 mm. The beam speed was variable and controlled by the machine's speed function. During fabrication, the powder bed temperature was maintained between 650 and 700 °C, whereas the melt pool temperature exceeded 1900 °C, ensuring stable melting conditions and high material densification.

Manufacturing was performed in accordance with the ISO 13485 quality management standard. The build process was conducted automatically under controlled vacuum conditions, followed by a cooling stage that lasted for several hours. The produced implant exhibited a relative density exceeding 99.5%, indicating a fully consolidated and homogeneous structure. The mechanical properties of the cranioplasty complied with the requirements specified for surgical implants in the standard ISO 5832-3, which include a minimum yield strength of 780 MPa, an ultimate tensile strength exceeding 860 MPa, and elongation at fracture greater than 10%.

### 2.3 Post-Processing

**Figure 3:**
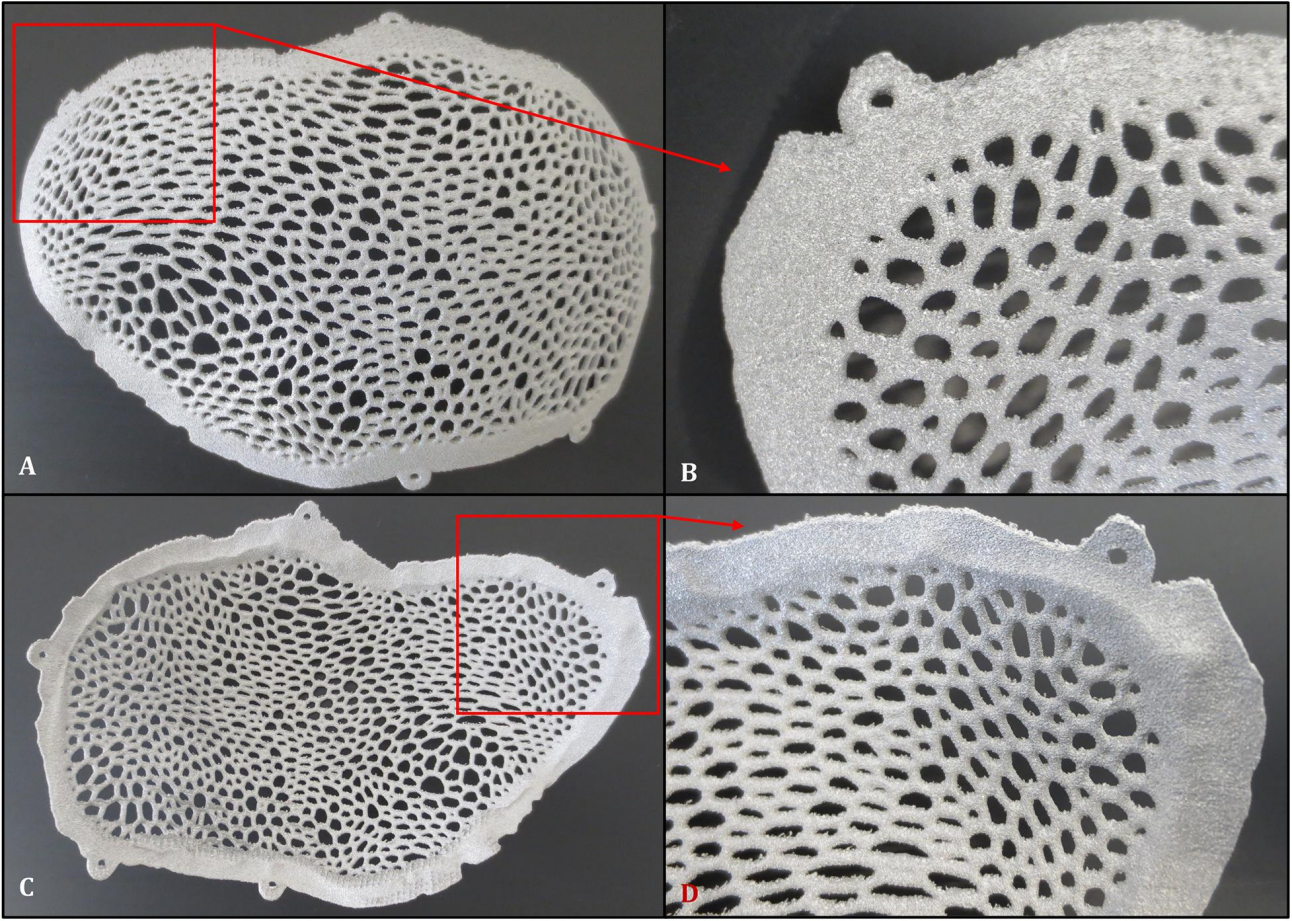
Rough surface appearance of the cranioplasty plate after three-dimensional fabrication using the EBM technique, prior to surface finishing of the implant: **(A)** external surface appearance of the plate. **(B)** detailed view of the anterior frontoparietal region of the external surface. **(C)** internal surface appearance of the plate. **(D)** detailed view of the anterior frontoparietal region of the internal surface.

Because the process was performed at a preheated powder bed temperature of 650–700 °C, residual stresses were significantly reduced, and no additional stress-relief heat treatment was applied. After fabrication, the residual powder was eliminated by blasting in a blast cabinet using titanium powder from the same batch, allowing powder reuse. Subsequently, the implant was cut from the build plate, and the support structures were manually removed using pliers. The cranial implant then underwent manual finishing. Due to the inherently rough surface texture of EBM-fabricated titanium components as shown in Figure 3, mechanical finishing was performed on both the external (exocranial) and internal (endocranial) surfaces using rotary instruments commonly employed for metallic implant finishing (15). The surface was further cleaned and refined using solid carbide mills suitable for titanium alloys, along with other polishing tools. A second blasting step with titanium powder was applied to remove debris generated during grinding. Dimensional control during finishing was ensured through continuous visual inspection of the grinding process, with material removal limited to the elimination of the as-built surface roughness. The process was carried out by trained personnel following established work instructions, and caliper measurements were used as an auxiliary tool to maintain dimensional accuracy.

The theoretical implant weight, estimated based on a volume of 12.06 cm^3^ and a relative density of 99.5%, was approximately 53.15 g, while the weight of the manufactured and post-processed implant was 39.87 g, as determined using an analytical balance (Kern EMS).

**Figure 4:**
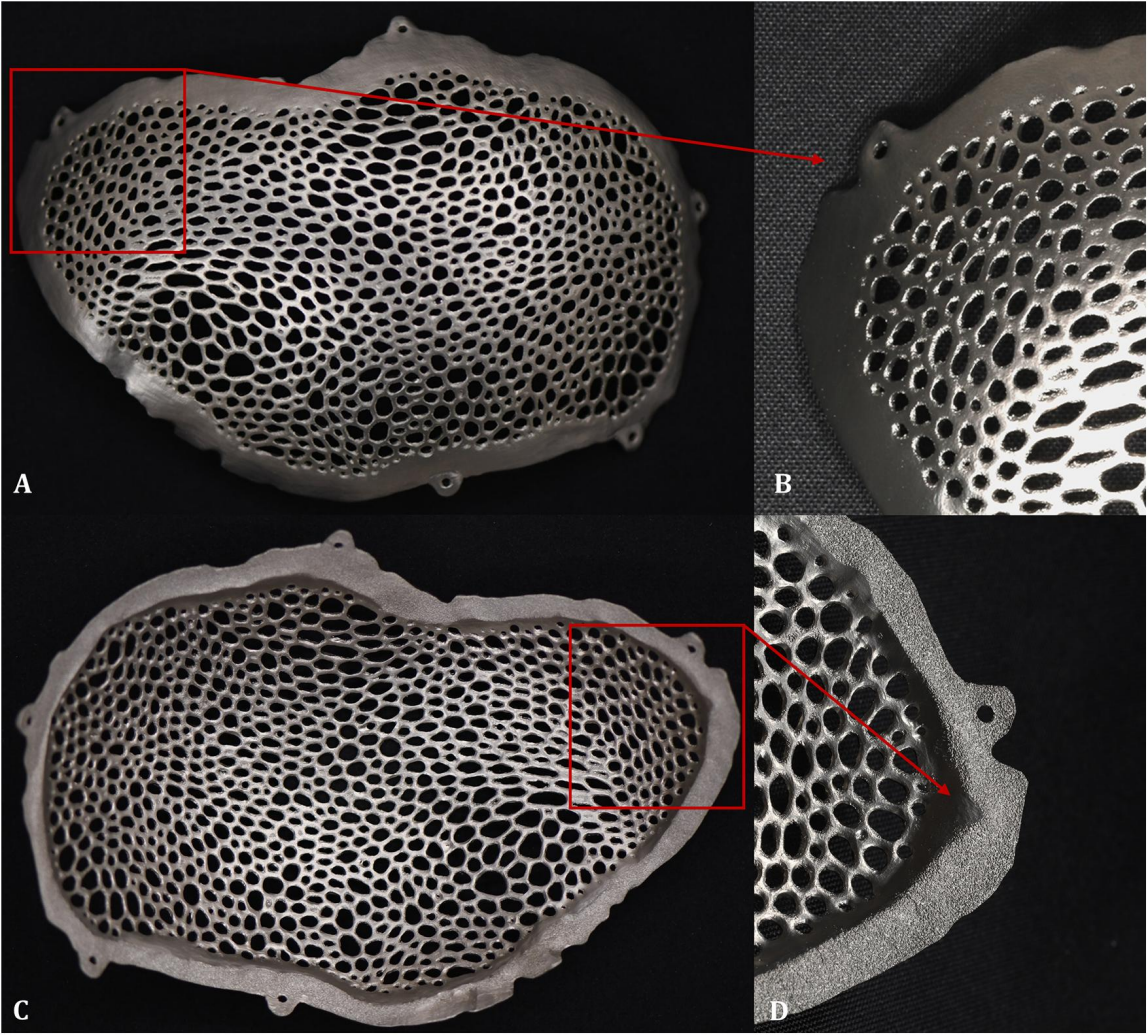
Appearance of the cranioplasty plate after surface finishing: **(A)** external surface of the plate. **(B)** detailed view of the anterior frontoparietal region of the external surface. **(C)** internal surface appearance of the plate. **(D)** detailed view of the anterior frontoparietal region of the internal surface, highlighting the marginal surface in contact with the bone, which was intentionally preserved as rough.

The peripheral region of the internal surface corresponding to the implant–bone contact interface was intentionally left unpolished to preserve the surface roughness, which may be favorable for bone–implant interaction. Attention was paid to maintaining the geometric integrity of the fixation flanges and screw holes during post-processing, as these features are critical for accurate intraoperative positioning and fixation. The external (exocranial) and internal (endocranial) surfaces, along with the accompanying details, are depicted in Figure 4.

### 2.4 Surface roughness

Surface roughness of the EBM-manufactured implant was measured before and after post-processing using a contact profilometer (SJ-2010, Mitutoyo Co., Japan). Ten measurements were performed on the outer boundary contour of the implant for each condition. The roughness parameters Ra, Rq, and Rz were determined in accordance with ISO 4287. The parameter Ra represents the arithmetic mean of the absolute deviation of the roughness profile from the mean line. Rq (root mean square roughness) corresponds to the quadratic mean of the profile deviations. The parameter Rz represents the average value of the maximum peak-to-valley heights measured within the sampling length of the surface profile. The mean values of each roughness parameter and their standard deviations are presented in Table 2.

**Table 2:** Surface roughness of cranioplasty (mean ± standard deviation).

Cranioplasty condition	Roughness, Ra [μm]	Roughness, Rq [μm]	Roughness, Rz [μm]	Surface profile recorded
As-built	19.41 ± 0.85	23.30 ± 0.52	83.28 ± 1.47	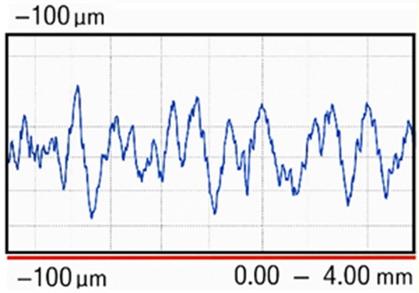
After finishing	1.43 ± 0.28	1.71 ± 0.35	6.31 ± 0.61	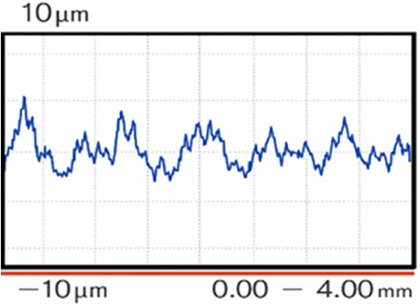

In the as-built condition, the implant exhibited a relatively rough surface with Ra = 19.41 µm, Rq = 23.30 µm, and Rz = 83.28 µm. The recorded surface profile showed variations within a range of ± 100 µm. After finishing the implant surfaces, the roughness decreased significantly to Ra = 1.43 µm, Rq = 1.71 µm, and Rz = 6.31 µm, whereas the surface profile variations were reduced to approximately ±10 µm. Overall, the Ra value of the surface roughness decreased by more than 90% after the finishing procedure.

### 2.5 Micro-Computed tomography digitization

To enable comprehensive dimensional analysis, the cranial implant was digitized in two distinct stages of the production workflow:

1. Immediately after EBM fabrication, prior to surface polishing,
2. After completion of post-processing and surface polish.

Digitization was performed using a high-resolution micro-computed tomography (micro-CT) system (Diondo d2, XWT-240-CT/UC240) operating at a spatial resolution of 76 µm. Given the voxel size of 76 µm, dimensional deviations below approximately one voxel should be interpreted as indicative trends rather than absolute dimensional certainty, as they may be influenced by image segmentation and surface interpolation effects. Micro-CT scanning was selected over optical surface scanning techniques because of the complex geometry of the Voronoi-based implant, which includes narrow openings and reflective metallic surfaces that may limit accurate surface acquisition using optical methods (12, 13).

**Figure 5:**
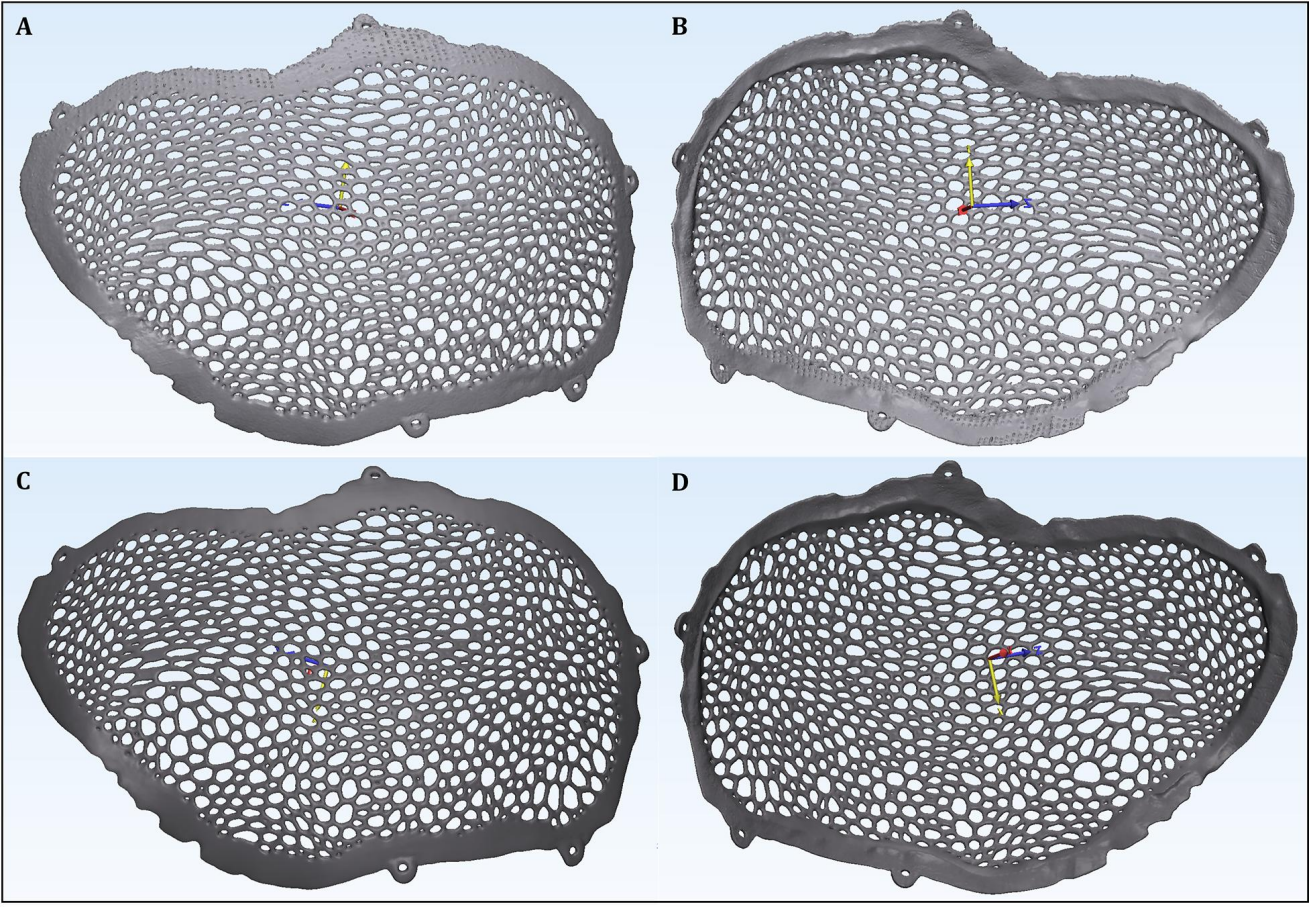
The two STL models of the plate scanned before and after surface finishing: **(A)** STL model of the unpolished plate (external surface). **(B)** STL model of the unpolished plate (internal surface). **(C)** STL model of the polished plate (external surface). **(D)** STL model of the polished plate (internal surface).

The acquired datasets were converted into stereolithographic (STL) models representing the unpolished cranial plate (Figures 5A,B) and the post-processed, polished cranial plate (Figures 5C,D). These STL models were subsequently used for dimensional comparisons with the original virtual implant design.

### 2.6 Dimensional accuracy assessment workflow

Quantitative dimensional accuracy analysis was performed using a dedicated three-dimensional metrology software (GOM Inspect, GOM GmbH, ZEISS Group, Braunschweig, Germany). The original virtual implant STL model, STL model of the unpolished implant, and STL model of the polished implant were imported into the software for comparative analyses.

Prior to the deviation analysis, an initial pre-alignment was applied to establish the approximate correspondence between the datasets within a common coordinate system. Given the complex and non-periodic geometry of the implant, a local best-fit alignment strategy was employed to minimize the global surface deviation while avoiding the introduction of artificial geometric constraints.

A local best-fit alignment was selected to reflect the clinically relevant adaptation of the implant surface to the reconstructed cranial contour, rather than enforcing artificial datum constraints that may not be preserved during surgical implantation. This approach prioritizes the global surface conformity of the patient-specific implant, which is considered more representative of intraoperative implant adaptation than alignment strategies based on predefined reference features.

Dimensional deviations were evaluated using full-field surface comparisons and visualized using color-coded deviation maps. A tolerance range of ±0.50 mm was selected for all analyses, reflecting the dimensional ranges commonly reported in the literature for patient-specific cranial implant fit (9).

Three comparative analyses were conducted:

1. Original virtual model vs. the unpolished implant model (assessment of additive-manufacturing-induced deviations)
2. Original virtual model vs. the polished implant model (assessment of cumulative manufacturing and postprocessing effects)
3. Unpolished implant model vs. polished implant model (assessment of post-processing-induced deviations)

In addition to full-field visualization, a quantitative analysis was performed by sampling more than 100 discrete measurement points distributed across the implant surface, including the central regions, Voronoi struts, mesh openings, peripheral contact zones, and fixation features. Spatial distribution descriptors, including mean surface deviation, deviation spread, and extreme values, were calculated to characterize the within-implant geometric variation, rather than the population-level statistical behavior.

## 3 Results

### 3.1 Geometric characteristics of the virtual reference model

**Figure 6:**
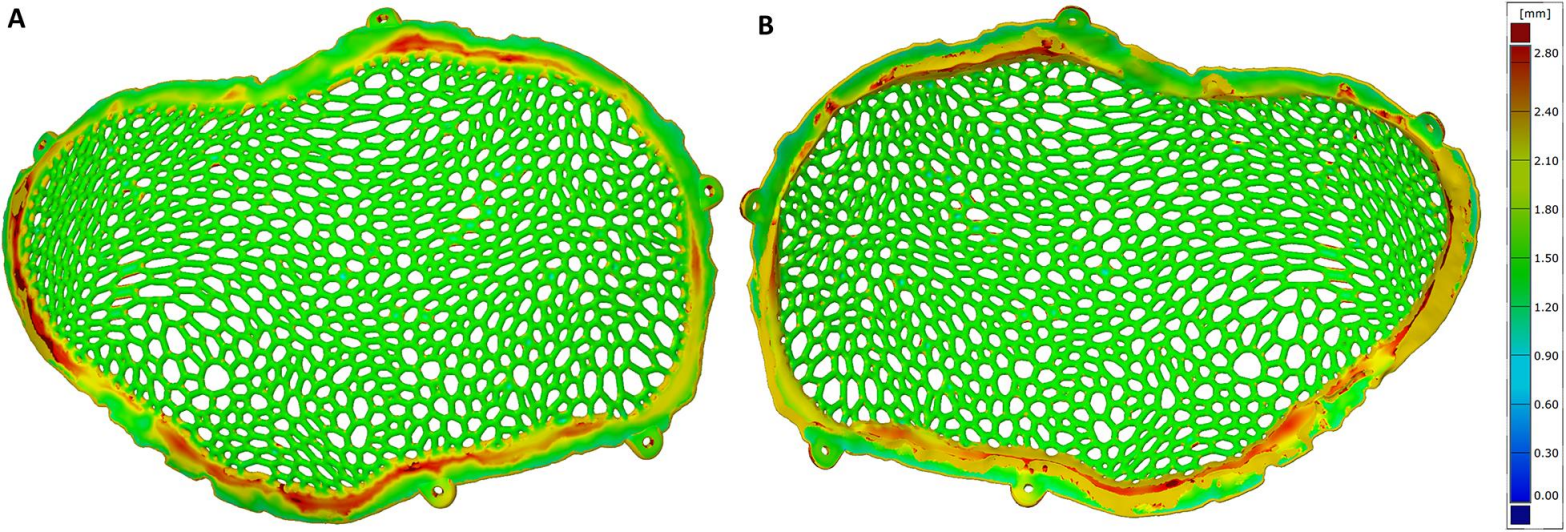
Diagram of the thickness analysis of the virtual (original) model of the personalized voronoi cranial implant: **(A)** external (exocranial) surface view. **(B)** internal (endocranial) surface view.

The virtual reference model exhibited the following overall dimensions: length, 141.50 mm; width, 88.45 mm; height, 73.85 mm; and total volume, 12.06 cm^3^. The original virtual implant model was analyzed to quantify the wall thickness distribution across the structure. Thickness analysis revealed a heterogeneous distribution, as illustrated by the color-coded thickness map shown in Figure 6.

The minimum and maximum wall thickness measured in the model were 1.33 and 2.80 mm, respectively. The mean wall thickness was 1.43 mm, with a standard deviation of ±0.24 mm, reflecting localized peripheral thickening rather than strut uniformity. Regions along the periphery of the implant that were manually thickened during the design stage exhibited locally increased thickness values, reaching up to 2.91 mm.

In general, the peripheral contour of the implant demonstrated wall thickness values ranging between 1.80 and 2.80 mm, whereas the struts forming the Voronoi-based macrostructure exhibited thicknesses predominantly between 1.41 and 1.52 mm.

The virtual implant model served exclusively as the nominal reference geometry for subsequent dimensional deviation analyses.

### 3.2 Dimensional deviation between the virtual original model and the unpolished EBM-produced cranial implant

The dimensional accuracy of the additive manufacturing process was assessed by comparing the original virtual STL model with the STL model of the implant obtained immediately after Electron Beam Melting production, prior to post-processing. A full-field surface comparison resulted in color-coded deviation maps illustrating the local geometric differences between the two models, with green corresponding to a zero deviation.

**Figure 7:**
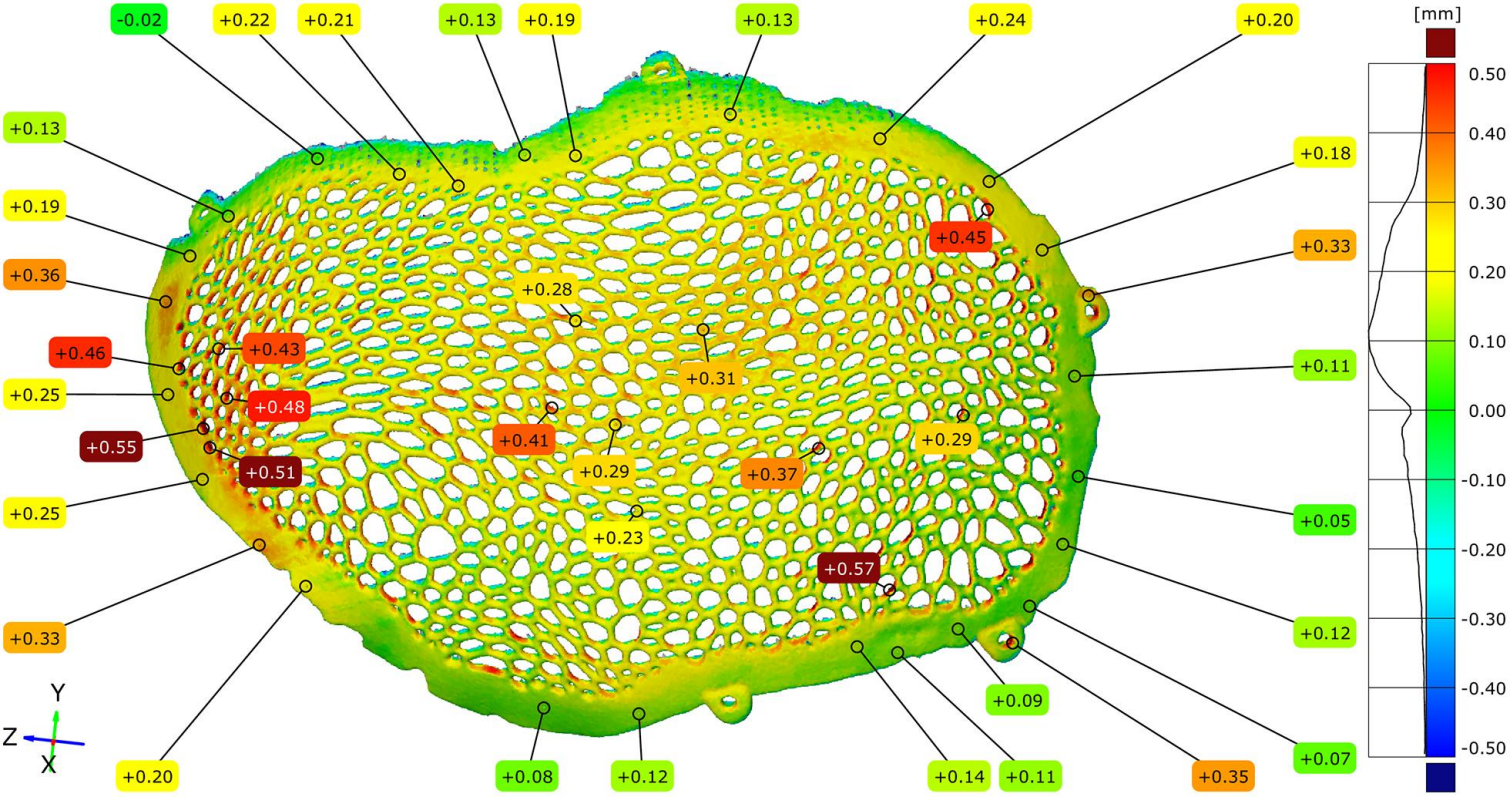
Comparative diagram between the original STL model and the unpolished model, highlighting the external (exocranial) surface of the implant.

**Figure 8:**
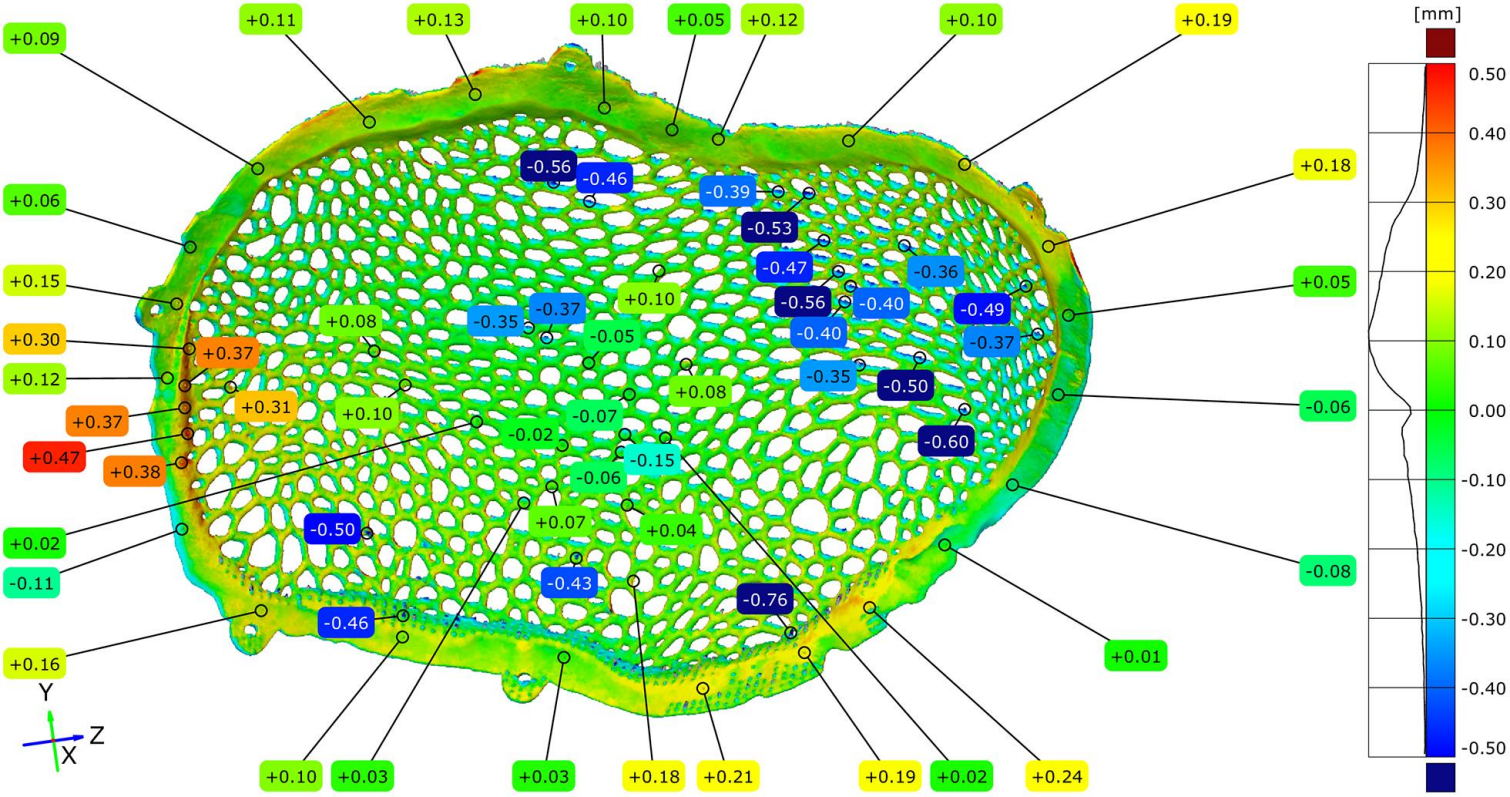
Comparative diagram between the original STL model and the unpolished model, highlighting the internal (endocranial) surface of the implant. Notice the contact points of the support structures on the inferior inner aspect of the cranial implant.

**Figure 9:**
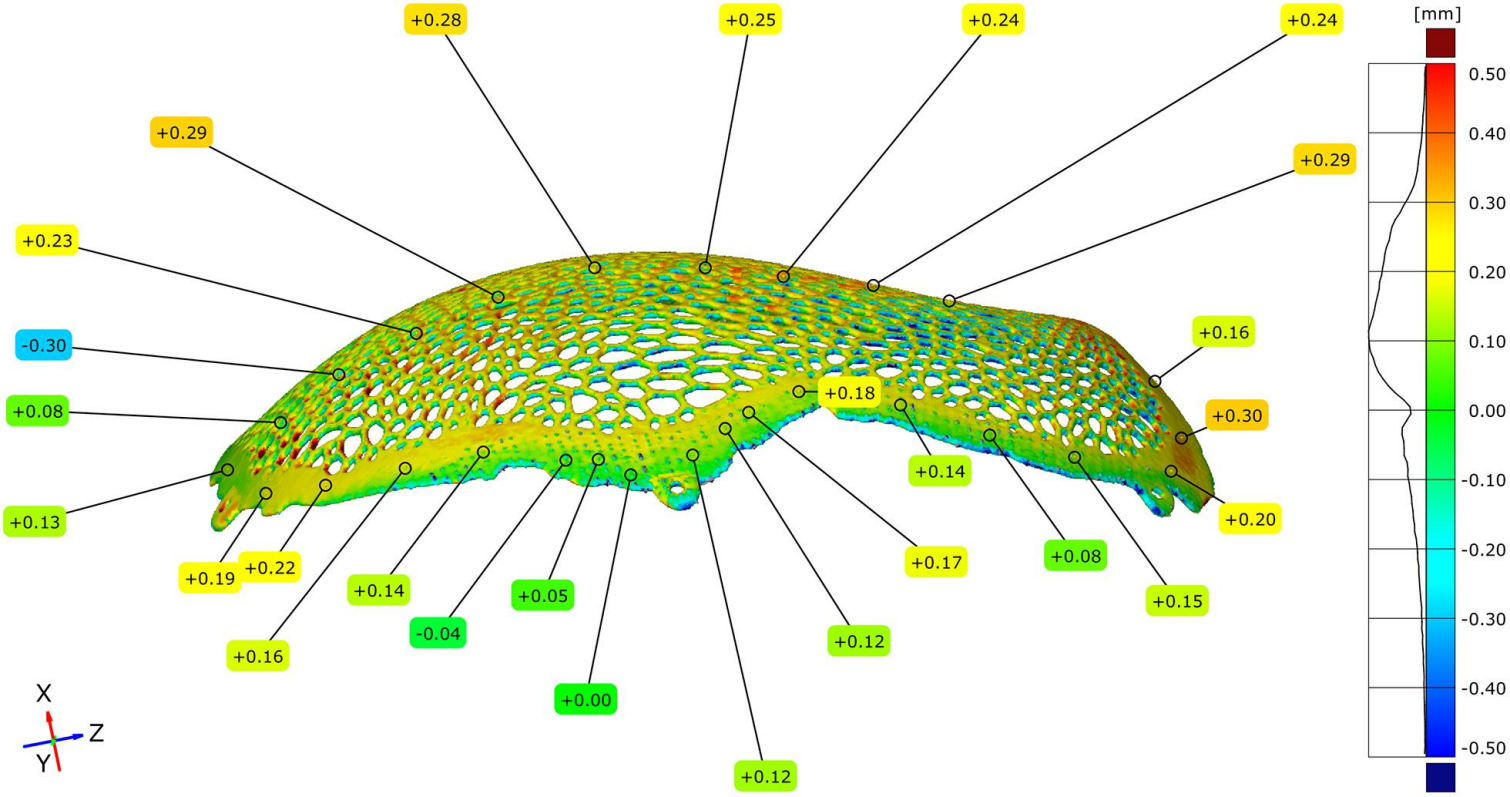
Comparative diagram between the original STL model and the model of the unpolished cranial plate – top view of the external (exocranial) implant surface where the contact points of the support structures are visible.

Across most of the implant surface, the dimensional deviations ranged between −0.20 mm and +0.35 mm. Localized regions exhibiting deviations below −0.45 mm were observed in areas associated with the support structure attachment. In addition, portions of the Voronoi strut network demonstrated positive deviations ranging from +0.43 mm to +0.55 mm, corresponding to the regions where partially sintered powder residues were present (Figures 7–9).

Histogram-based frequency analysis indicated that the most observed deviation ranges were distributed between +0.05 mm and +0.25 mm, with the highest concentration centered around +0.10 mm. To complement the full-field analysis, dimensional deviations were quantified at more than 100 discrete measurement points distributed across the external and internal implant surfaces, including the central regions, mesh openings, Voronoi struts, and peripheral contact zones.

Positive deviations were more frequently observed within the mesh openings, particularly on the external surface of the implant and in regions corresponding to the contact with support structures during fabrication. Negative deviations were identified primarily on the internal surface of the implant and along the peripheral contact zones, where localized thinning was consistent with the tangential implant–bone interface planned in the design.

### 3.3 Dimensional deviation between the virtual original model and the polished implant

To assess the cumulative geometric effects of additive manufacturing and post-processing, the original virtual STL model was compared with the STL model of the implant following the completion of surface finishing. Full-field deviation analysis revealed that most dimensional deviations were negative, ranging from −0.38 mm to −0.06 mm, with the highest concentration centered around −0.18 mm. These deviations can be partially explained by the reduction in implant mass, as the theoretical weight was estimated at 53.15 g, while the manufactured and post-processed implant had a measured weight of 39.87 g.

**Figure 10:**
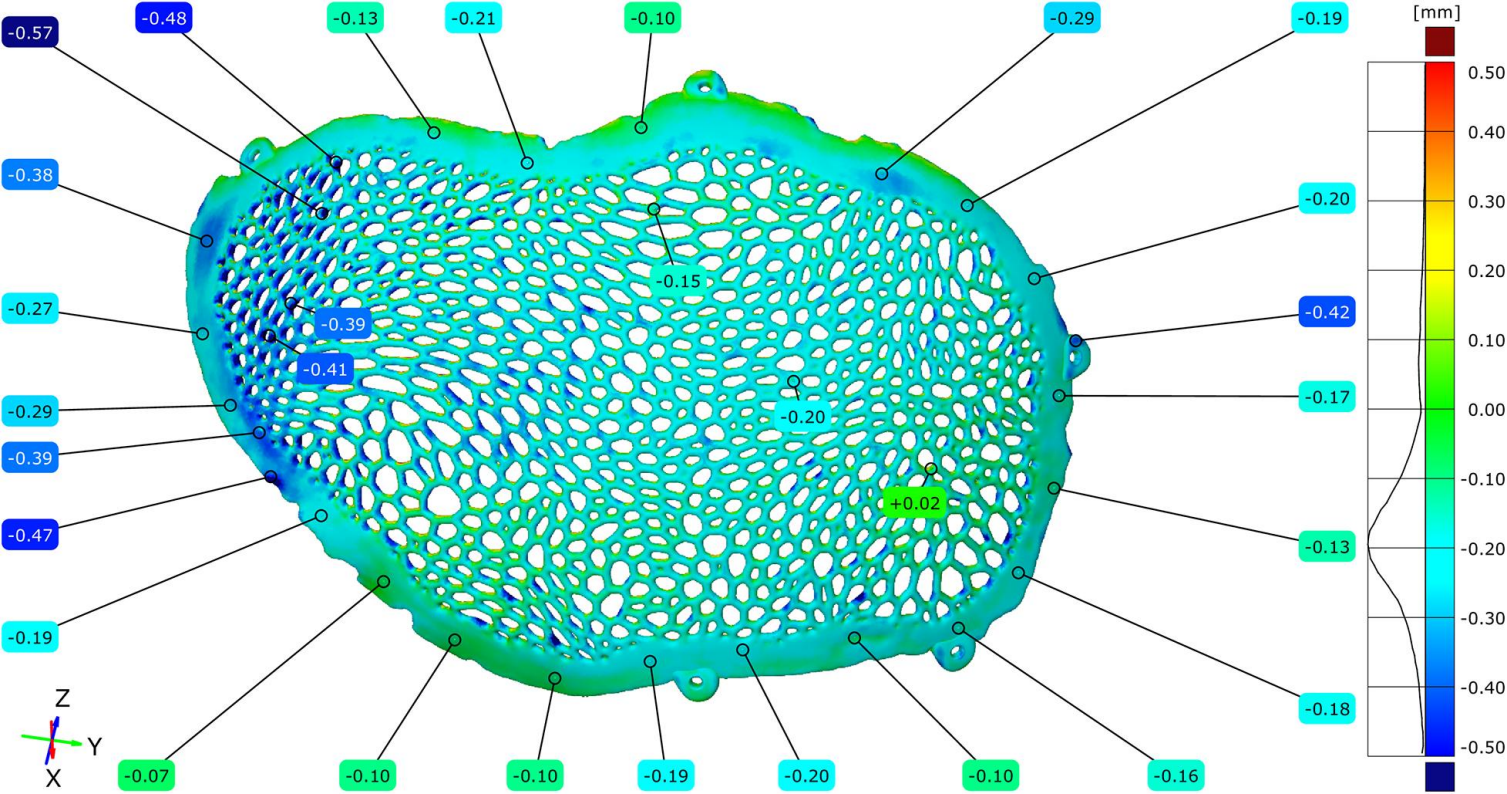
Comparison diagram between the original STL model and the polished STL model – external implant surface.

**Figure 11:**
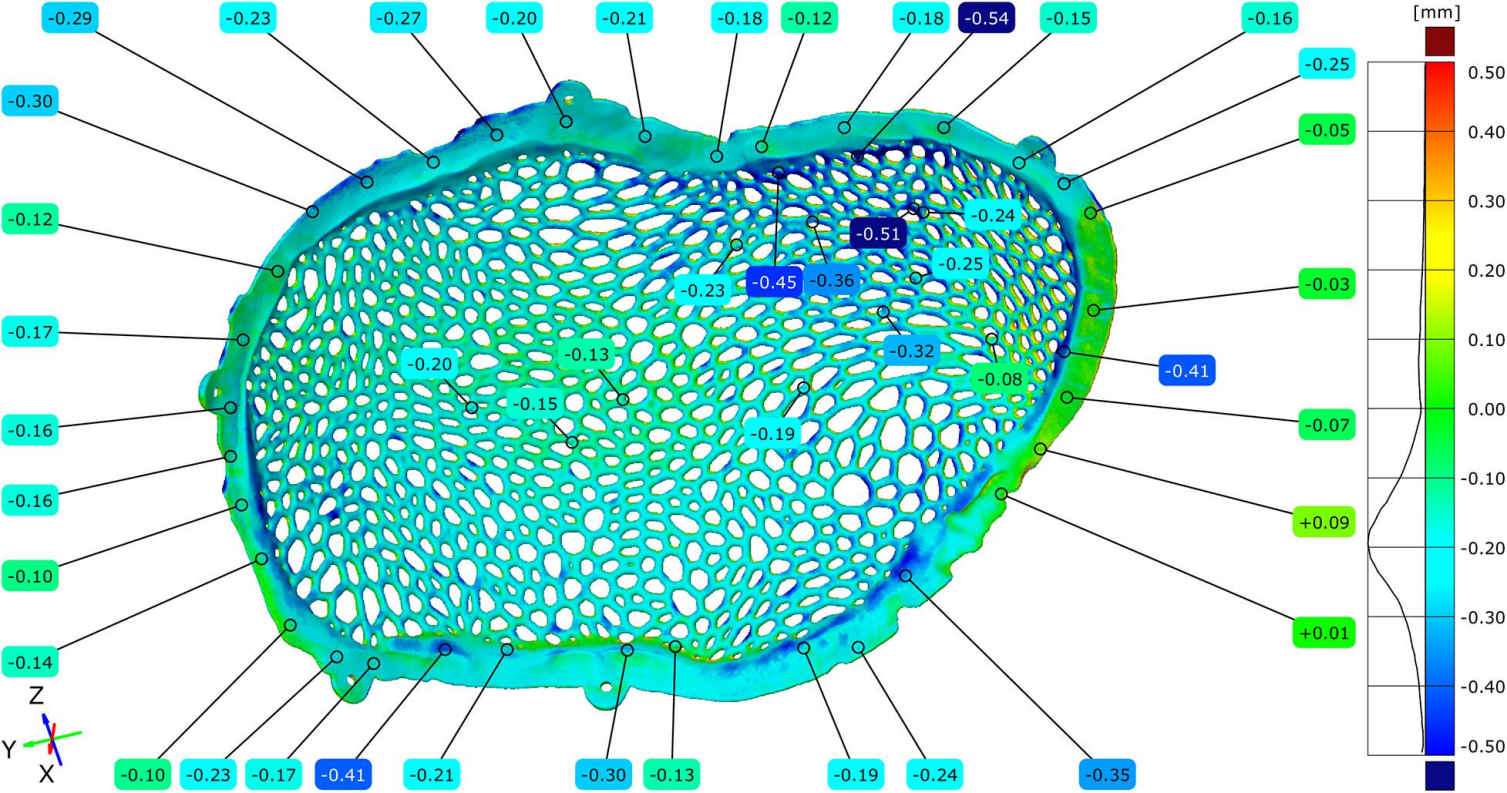
Comparison diagram between the original STL model and the polished STL model - internal implant surface.

**Figure 12:**
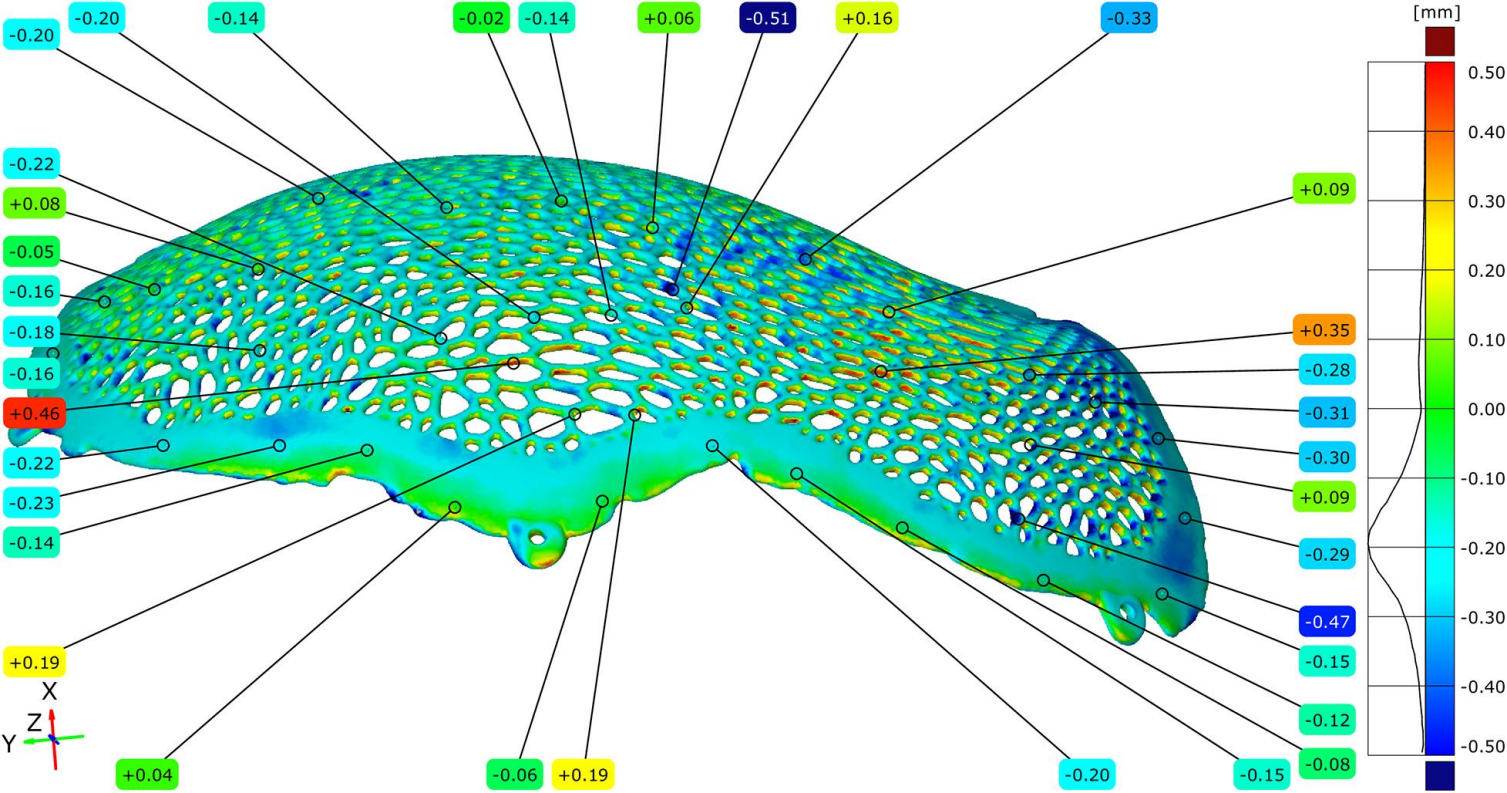
Comparison diagram between the original STL model and the polished STL model, top view of the external (exocranial) implant surface–notice the total removal of the contact points of the support structures.

Localized deviations between −0.41 and −0.54 mm were observed in portions of the Voronoi strut network. Quantitative point-based analysis performed at more than 100 locations across the implant surface confirmed the predominance of negative deviation values (Figures 10–12).

### 3.4 Dimensional deviation induced by post-processing

Geometric deviations attributable specifically to post-processing were isolated by directly comparing the STL model of the unpolished implant with the STL model of the polished implant. The full-field deviation maps demonstrated predominantly negative deviations across the polished regions.

**Figure 13:**
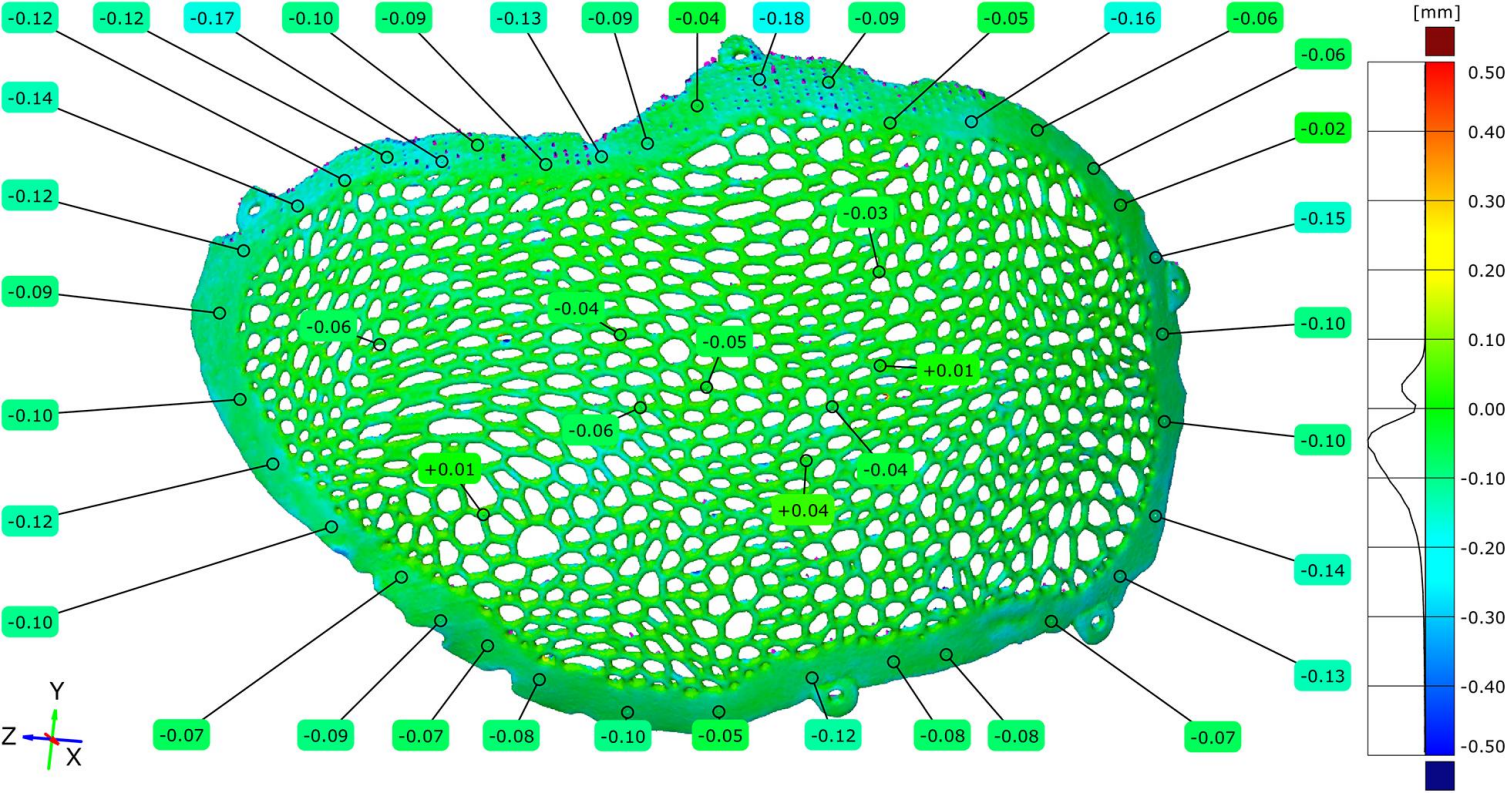
Comparative metrological analysis between the STL model of the unpolished plate and the STL model of the polished plate - external implant surface.

**Figure 14:**
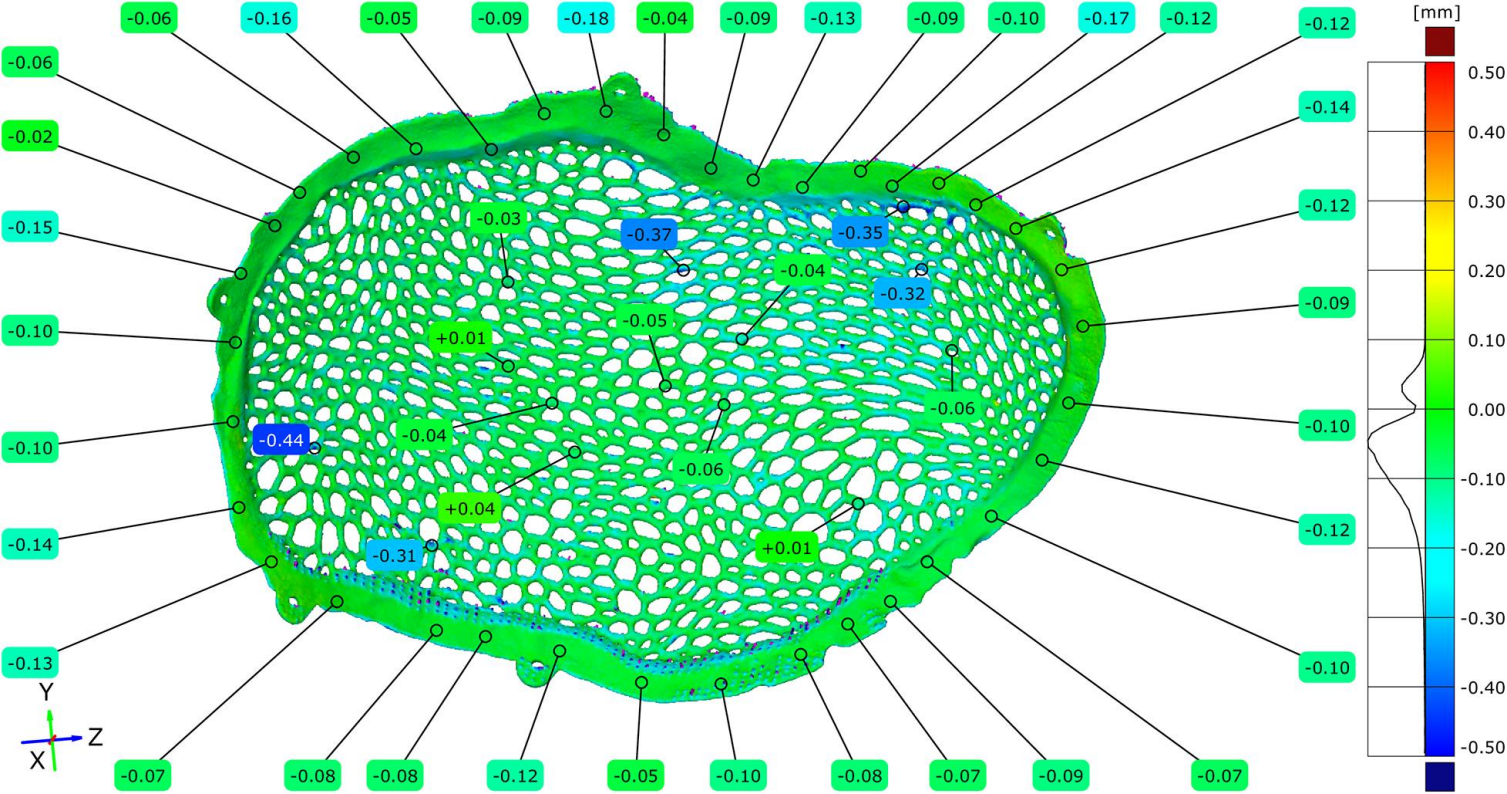
Comparative metrological analysis between the STL model of the unpolished plate and the STL model of the polished plate - internal implant surface.

**Figure 15:**
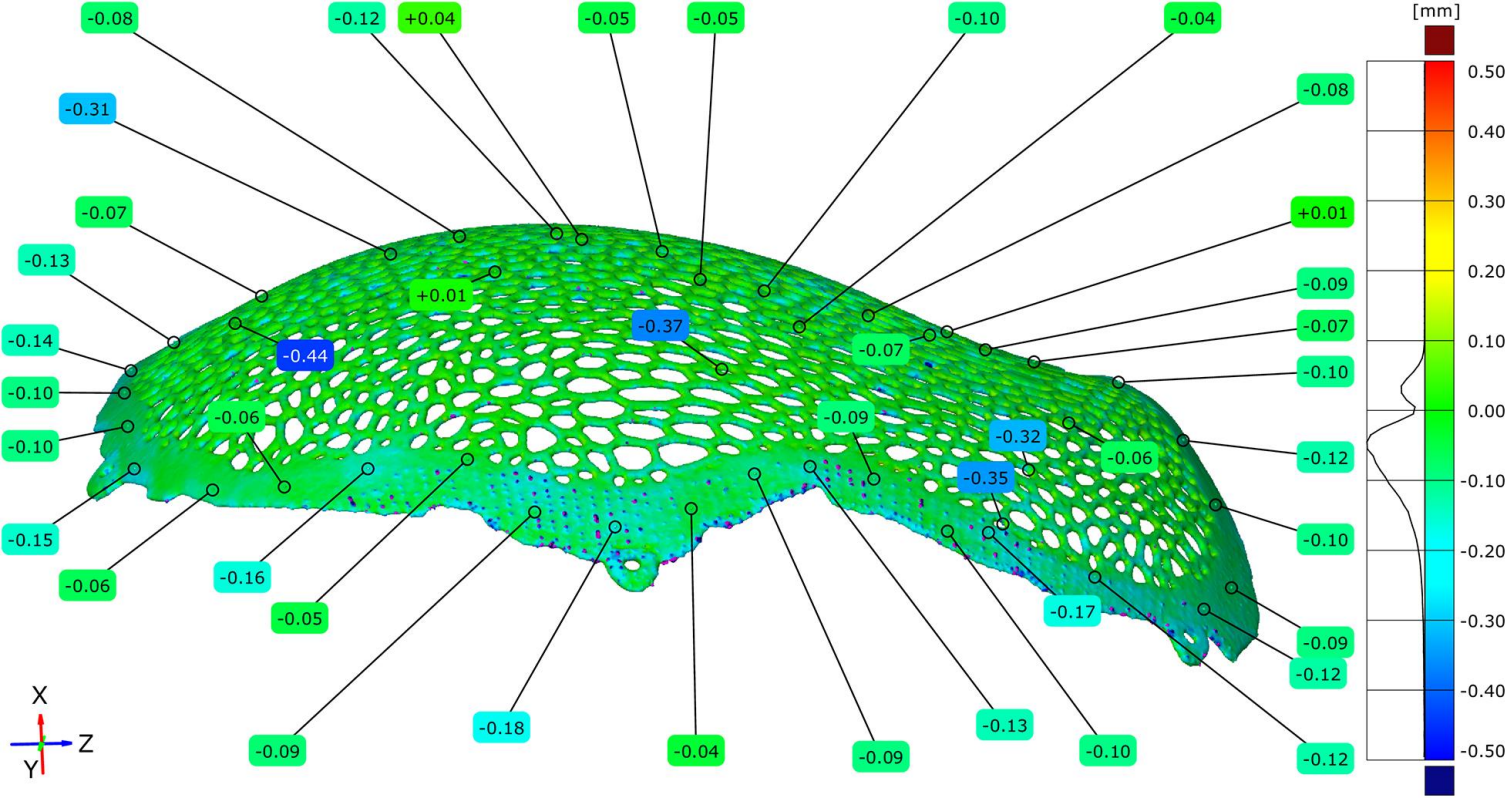
Comparative metrological analysis between the STL model of the unpolished plate and the STL model of the polished plate - top view of the external (exocranial) implant surface, where the contact points of the support structures are visible.

The maximum positive deviation measured was +0.04 mm, while the maximum negative deviation reached −0.18 mm. The mean deviation across the implant surface was −0.05 mm (Figures 13–15).

To facilitate the quantitative interpretation of the effect of surface finishing, a relative reduction in dimensional deviation was calculated based on the maximum positive deviation values observed before and after post-processing. In unpolished conditions, the maximum positive deviation recorded during the full-field comparison between the virtual model and the manufactured implant reached +0.55 mm, which was mainly associated with powder adhesion and support interaction. After post-processing, the maximum positive deviation decreased to +0.04 mm. The relative reduction in the positive deviation was calculated using the following formula:

Reduction (%) = (Dinitial−Dfinal)/Dinitial × 100

where Dinitial represents the maximum positive deviation measured in the unpolished condition and Dfinal represents the maximum positive deviation after surface finishing. Based on these values, post-processing resulted in an approximately 93% reduction in the maximum positive deviation.

In addition, the magnitude of the average deviation was evaluated to estimate the overall geometric correction introduced by surface finishing. The most frequently observed deviation range in unpolished condition was centered around approximately +0.10 mm, whereas the mean deviation measured after post-processing was −0.05 mm. Considering the absolute deviation magnitudes, this corresponds to an approximate 50% reduction in mean deviation magnitude, indicating a systematic improvement in geometric conformity following finishing. Quantitative point-based analysis confirmed that negative deviations were consistently distributed across the areas subjected to mechanical finishing.

Regions previously associated with support structures exhibited reduced surface irregularities following post-processing, accompanied by limited negative deviations ranging from −0.04 to −0.18 mm. The overall topology of the Voronoi-based macrostructure remained continuous, with no evidence of strut discontinuity or geometric collapse during the entire process.

## 4 Discussion

This investigation should be interpreted as a methodological proof-of-concept that demonstrates the feasibility of a multistage dimensional accuracy assessment workflow for patient-specific cranial implants fabricated using EBM. The primary objective of this study was not to establish population-level manufacturing accuracy or clinical performance but to demonstrate a reproducible micro-CT-based metrological framework capable of differentiating geometric deviations introduced during additive manufacturing and subsequent post-processing. Accordingly, the dimensional accuracy of a patient-specific cranial implant featuring a complex Voronoi-based macrostructure was evaluated as a geometrically demanding test case, focusing on process validation rather than design innovation.

### 4.1 Feasibility of manufacturing complex cranial implant geometries using EBM

The successful fabrication of patient-specific cranial implants without macroscopic deformation or build failure demonstrates that EBM can accommodate geometrically demanding implant architectures, including thin interconnected struts, multiple openings, and nonperiodic Voronoi-based networks (18). These findings are consistent with previous reports describing the capability of EBM to fabricate dense Ti–6Al–4 V cellular and porous structures for biomedical applications (10).

From a process perspective, EBM is conducted under high-vacuum conditions and involves powder bed preheating and partial sintering prior to full melting; these process characteristics contribute to improved thermal stability during fabrication (19, 20). These attributes are particularly relevant for cranial implants, where large surface areas, curvature, and locally unsupported features may increase susceptibility to thermal distortion. In the present study, the fixation flanges and screw-hole regions preserved their intended geometry, indicating that functionally critical features can be manufactured using EBM.

Previous feasibility studies on Voronoi-based cranial implants fabricated using laser powder bed fusion technologies have highlighted challenges related to overhanging features, sharp junctions, and support structure requirements (17, 21). Although the present study did not provide a direct comparative analysis of powder bed fusion technologies, the observed fabrication outcomes suggest that EBM is a robust alternative for producing patient-specific cranial implants with complex macrostructures.

### 4.2 Dimensional accuracy after EBM fabrication

Quantitative micro-computed tomography–based comparison between the virtually planned implant model and the implant geometry immediately after EBM fabrication revealed dimensional deviations predominantly within a ± 0.50 mm range. The positive deviations observed along portions of the Voronoi strut network are consistent with partial powder adhesion and sintering effects, phenomena that have been previously reported in the powder bed fusion of lattice and porous structures (12–14).

Negative deviations were primarily localized in the peripheral regions and areas associated with the support structure contact and subsequent removal. The fixation regions exhibited limited deviation relative to the planned geometry, which is particularly relevant for intraoperative handling and implant fixation. These findings support the suitability of EBM for manufacturing patient-specific cranial implants with dimensional fidelity comparable to that reported for craniofacial implants fabricated using alternative additive manufacturing workflows (9).

Rather than proposing a universal tolerance threshold, the present findings align with published dimensional accuracy assessments of patient-specific craniofacial implants, which commonly report deviations in the sub-millimeter to approximately 1 mm range, depending on the implant geometry, manufacturing method, and evaluation strategy (9, 22). Within this context, the dimensional fidelity observed following EBM fabrication supports the clinical relevance of the quantitative accuracy validation for complex cranial implant geometries.

### 4.3 Influence of post-processing on dimensional fidelity

Surface finishing resulted in a systematic shift toward negative dimensional deviations when the polished implant was compared with the original virtual model. The direct comparison between the unpolished and polished implant geometries enabled the isolation of deviations attributable specifically to post-processing, revealing a controlled material removal characterized by a mean deviation of −0.05 mm and a maximum negative deviation of −0.18 mm. These deviations are consistent with the reduction in implant mass, from a theoretical 53.15 g to a measured 39.87 g. Moreover, considering the overall dimensions of the cranial implant (length: 141.50 mm; width: 88.45 mm; height: 73.85 mm), the observed dimensional deviations can be considered acceptable.

In general, state-of-the-art PBF systems achieve dimensional accuracies of approximately ±0.20 mm, with an additional deviation of ±0.10 mm per 100 mm. However, this level of accuracy is often insufficient to meet strict geometric dimensioning and tolerancing requirements, and therefore post-processing operations, such as CNC machining or manual finishing, are typically required to achieve the desired precision. For complex and large components, dimensional deviations may be even more pronounced. For instance, Müller et al. reported deviations of up to ±0.30 mm for an additively manufactured AlSi10Mg wheel carrier (23), while studies conducted at CERN (Switzerland) on superconducting radio-frequency components demonstrated accuracies of approximately ±0.20 mm (24).

In the field of biomedical applications, including facial and orthopedic titanium implants, post-processing has been shown to significantly improve surface roughness and homogeneity, while maintaining dimensional deviations typically within ±0.12 mm (25). The deviations observed in the present study are consistent with these findings, confirming that controlled finishing processes can achieve acceptable dimensional accuracy for patient-specific cranial implants.

These observations are consistent with prior studies demonstrating that post-processing procedures can significantly influence the final dimensions of additively manufactured lattices and porous structures, even when the global geometry is preserved (15). Although surface finishing is frequently required to reduce surface roughness and remove residual support structures, it is a critical step that should be considered during virtual planning, particularly for implants incorporating thin struts or functionally relevant interfaces (26).

Despite the localized material removal, the overall topology and structural continuity of the Voronoi-based macrostructure were preserved following post-processing. This indicates that surface finishing does not necessarily compromise the geometric integrity of complex implant architectures fabricated using EBM when appropriately controlled.

### 4.4 Clinical interpretation and relevance

From a clinical perspective, the dimensional deviations observed across all three comparative analyses are unlikely to compromise implant adaptation or fixation, as they remain within the accuracy range reported for patient-specific craniofacial implants (8, 9). The peripheral contact regions maintained geometric continuity, and the fixation features preserved their intended dimensions, thereby supporting the practical applicability of the proposed manufacturing and validation workflow.

It is important to emphasize that the Voronoi-based macrostructure evaluated in this study was not introduced as a novel design concept but rather as a geometrically demanding test case for manufacturing validation. The heterogeneous thickness distribution inherent to such architectures may further mitigate the clinical impact of localized deviations, because the reinforced peripheral regions provide a degree of tolerance against minor dimensional variations.

### 4.5 Limitations and future perspectives

This study has several limitations. First, dimensional analysis was performed on a single patient-specific implant geometry, and broader validation across multiple defect types, sizes, and anatomical locations is required to generalize the findings. Second, this study focused exclusively on dimensional accuracy and did not include mechanical testing or biological evaluation, both of which are necessary to fully characterize implant performance.

Since the finishing procedure directly affects the dimensional deviations of complex surfaces fabricated by metal additive manufacturing, and the final surface roughness and geometric accuracy of cranioplasty implants depend on the skills and experience of technicians performing manual finishing, future work will focus on the implementation of automated finishing techniques, such as electromechanical polishing and drag finishing for EBM-processed implants (27).

Future studies should expand this validation framework to include larger case series, integrate mechanical and fatigue testing, and explore alternative post-processing strategies. Comparative investigations evaluating identical cranial implant geometries fabricated using different powder bed fusion technologies may further elucidate the relative advantages and limitations of EBM for patient-specific cranioplasty applications.

## 5 Conclusions

This study demonstrated the feasibility of manufacturing a patient-specific cranial implant featuring a complex Voronoi-based macrostructure using EBM and quantitatively assessed its dimensional fidelity through a multistage micro-CT–based workflow. In this way, a lightweight cranioplasty implant made of titanium alloy was successfully fabricated. Full-field comparisons between the virtual design, the unpolished implant, and the post-processed implant showed that the geometric deviations introduced during additive manufacturing and subsequent surface finishing were spatially consistent and remained within the ranges reported in the literature for patient-specific cranioplasty applications.

The findings indicate that EBM can accommodate demanding non-periodic cranial implant architectures while preserving dimensional fidelity in functionally relevant regions, including peripheral contact zones and fixation features. Controlled post-processing resulted in systematic material removal without compromising the structural continuity of the Voronoi-based macrostructure.

Although the implant geometry was derived from a previously reported design workflow, this study provides a focused validation of the EBM manufacturing and post-processing pipeline using a reproducible multistage dimensional accuracy assessment methodology. This framework enables differentiation between manufacturing- and post-processing-induced geometric deviations and supports robust digital-to-physical workflows for patient-specific cranial implant fabrication.

Future investigations should expand this validation approach to larger implant cohorts and integrate mechanical and biological performance assessments to further substantiate the translational applicability of complex cranial implants produced via Electron Beam Melting.

## Data Availability

The data analyzed in this study is subject to the following licenses/restrictions: The original contributions presented in the study are included in the article, further inquiries can be directed to the corresponding author. Requests to access these datasets should be directed to Daniel Ostas, daniel.ostas@gmail.com.
